# Current Therapeutic Landscape and Safety Roadmap for Targeting the Aryl Hydrocarbon Receptor in Inflammatory Gastrointestinal Indications

**DOI:** 10.3390/cells11101708

**Published:** 2022-05-21

**Authors:** Samantha C. Faber, Tejas S. Lahoti, Ewan R. Taylor, Lauren Lewis, Jessica M. Sapiro, Vicencia Toledo Sales, Yvonne P. Dragan, Brandon D. Jeffy

**Affiliations:** 1 Discovery Toxicology, Drug Safety Research & Evaluation, Takeda Development Center Americas, Inc., San Diego, CA 92121, USA; tejas.lahoti@takeda.com (T.S.L.); yvonne.dragan@takeda.com (Y.P.D.); brandon.jeffy@takeda.com (B.D.J.); 2 Gastrointestinal Immunology, Gastrointestinal Drug Discovery Unit, Takeda Development Center Americas, Inc., San Diego, CA 92121, USA; ewan.taylor@takeda.com; 3 Investigative Toxicology, Drug Safety Research & Evaluation, Takeda Development Center Americas, Inc., Cambridge, MA 02139, USA; lauren.lewis@bms.com (L.L.); vicencia.toledo-sales@takeda.com (V.T.S.); 4 Regulatory Toxicology, Drug Safety Research & Evaluation, Takeda Development Center Americas, Inc., Cambridge, MA 02139, USA; jessica.sapiro@takeda.com

**Keywords:** aryl hydrocarbon receptor, CYP1A1, gastrointestinal toxicity, inflammation, PROTAC, oligonucleotides, microbiome, toxicogenomics, safety assessment

## Abstract

Target modulation of the AhR for inflammatory gastrointestinal (GI) conditions holds great promise but also the potential for safety liabilities both within and beyond the GI tract. The ubiquitous expression of the AhR across mammalian tissues coupled with its role in diverse signaling pathways makes development of a “clean” AhR therapeutically challenging. Ligand promiscuity and diversity in context-specific AhR activation further complicates targeting the AhR for drug development due to limitations surrounding clinical translatability. Despite these concerns, several approaches to target the AhR have been explored such as small molecules, microbials, PROTACs, and oligonucleotide-based approaches. These various chemical modalities are not without safety liabilities and require unique de-risking strategies to parse out toxicities. Collectively, these programs can benefit from in silico and in vitro methodologies that investigate specific AhR pathway activation and have the potential to implement thresholding parameters to categorize AhR ligands as “high” or “low” risk for sustained AhR activation. Exploration into transcriptomic signatures for AhR safety assessment, incorporation of physiologically-relevant in vitro model systems, and investigation into chronic activation of the AhR by structurally diverse ligands will help address gaps in our understanding regarding AhR-dependent toxicities. Here, we review the role of the AhR within the GI tract, novel therapeutic modality approaches to target the AhR, key AhR-dependent safety liabilities, and relevant strategies that can be implemented to address drug safety concerns. Together, this review discusses the emerging therapeutic landscape of modalities targeting the AhR for inflammatory GI indications and offers a safety roadmap for AhR drug development.

## 1. Introduction

Dysfunctional response of the immune system to environmental or dietary triggers can result in chronic inflammatory disorders of the gastrointestinal (GI) tract. For prevalent inflammatory GI diseases, such as inflammatory bowel disease (IBD) (including Crohn’s disease (CD) and ulcerative colitis (UC)), alterations in microbiota, exogenous or endogenous factors, and/or pathogenic infection within the gut microbiome influences immune function and potentiation of inflammation. Deficiency in mucosal immunity across epithelial, immune, and mucosal layers promotes intestinal permeability, or a “leaky gut”, which exacerbates inflammatory phenotypes and further decreases barrier defense. Identification of molecular targets and/or pathways that can restore barrier function and alleviate overt inflammatory signaling can aid in restoring tissue homeostasis within the gut microenvironment.

The aryl hydrocarbon receptor (AhR) is a ligand-activated basic helix-loop-helix (bHLH)-Per-aryl hydrocarbon receptor nuclear translocator (ARNT)-Sim (PAS)-containing transcription factor that acts as a xenosensor for detection of diverse metabolic, environmental, and dietary stimuli to regulate ligand-, cell-, and tissue-specific physiological, and/or toxicological effects. While initial interest in the AhR and AhR-dependent signaling focused on understanding distinct AhR-dependent molecular mechanisms driving a spectrum of dioxin-like compound (DLC) (i.e., 2,3,7,8-tetrachloro-*p*-dioxin (TCDD))-dependent toxicities [1], findings in transgenic AhR mouse knockout (AhR^−/−^) studies revealed a beneficial role for the AhR in regulation of innate and adaptive immunity within the gut [2,3]. AhR^−/−^ mice demonstrated diminished protection from immune-mediated barrier damage and/or permeability (e.g., gut, lung, skin, thymus, and urinary tract) [4,5,6], altered microbiome composition and homeostasis [7], increased intestinal metabolic stress [7], dose-dependent increases in drug-induced histological colon damage [8], and enhanced susceptibility to pathogens [9,10] relative to wild-type. Targeted AhR knockout within intestinal epithelial cells (IECs) (Vil1^CRE^AhR^fl/fl^) supported ligand- and AhR-dependent regulation of crypt intestinal stem cell differentiation, IEC regeneration, inhibition of pro-inflammatory signaling, and preservation of barrier integrity, while addition of *Citrobacter rodentium* to IEC-specific AhR^−/−^ resulted in dysregulation of intestinal stem cell proliferation, differentiation, and subsequent increased tumor burden relative to wild-type mice [11]. Exogenous (e.g., β-naphthoflavone (β-NF) and TCDD) and candidate endogenous AhR ligands found in the gut (e.g., tryptophan (Trp) metabolites) have been shown to mediate protective pathways within murine models of colitis [5,6] and promote AhR-dependent anti-inflammatory signaling via Il-10/Il-10rα, tight junction formation, and enhanced barrier integrity within IECs [12]. Notably, diminished intestinal AhR expression levels have been reported in patients with IBD compared to healthy volunteers [13], and synthetic AhR ligands have been shown to inhibit inflammation through induction of IL-22 mRNA and protein in T cells from patients with IBD in vitro [14]. Together, these findings suggest that the AhR is critical to intestinal homeostasis and indicates that therapeutic modulation of the AhR and/or AhR signaling pathway could combat various inflammatory GI diseases/disorders. The difficulty lies in balancing the dual nature of the AhR in disease biology and its role in DLC-mediated toxicity.

The specific AhR ligand used, cell type, or tissue involved, and spectrum of cytokines/chemokines modulated can influence AhR-dependent promotion or suppression of inflammation. This further confounds whether a given therapeutic modulator will induce AhR-dependent toxicological or physiological gene batteries and downstream effects. Therapeutic modulation of the AhR faces complex challenges that need to be addressed including (i) broad expression patterns across mammalian organ systems, (ii) development and regulation of immune cell populations that both confer and oppose tissue- and organ-specific autoimmunity, and (iii) limited understanding of the distinct underlying mechanisms of AhR-dependent beneficial or toxicological gene expression. AhR ligand-dependent and ligand-independent signal transduction pathways can have varied dimerization partners [15,16] (e.g., RelB subunit of nuclear factor kappa B (NF-κB) or Krüppel-like factor 6 (KLF6)), cellular crosstalk (e.g., nuclear hormone receptors and growth factors), co-activator, co-repressor, and/or enhancer recruitment, and species-specific and/or ligand-selective activity resulting in unique AhR-dependent gene signatures. Lack of a crystal structure of the AhR ligand binding domain limits accurate prediction of quantitative structure–activity relationships (SAR) for diverse AhR ligands (i.e., agonists, antagonists, and selective AhR modulators (SAhRMs) [17]), generation of structural alerts, and safety-by-design de-risking approaches within early drug discovery programs. De-risking potential safety liabilities associated with AhR modulation is further complicated by the advent of new therapeutic modalities, which still lack proper mechanistic understanding of on- versus off-target toxicity profiles. 

Despite these concerns, several groups have embarked on investigational and clinical studies focused on therapeutically modulating the AhR and downstream signaling pathway. Apart from traditional small molecule approaches, newer chemical modalities such as microbials, PROteolysis TArgeting Chimeras (PROTACs), and oligonucleotides have been explored to selectively target the promiscuous AhR and/or AhR signaling. In addition to developing a better understanding of the mechanism(s) of action for these new therapeutic modalities, thorough examination of modality-specific and AhR-dependent safety liabilities are necessary. Here, we highlight the role of the AhR in gut immunity, explore the current therapeutic landscape of new modality approaches targeting the AhR, and discuss safety liabilities and de-risking strategies that need to be considered before researchers can pharmacologically modulate the AhR with confidence for inflammatory GI diseases and disorders. 

## 2. Role of AhR within the Gut Microenvironment

Within the GI tract, AhR ligands can originate from several sources (e.g., commensal flora, xenobiotic exogenous/endogenous metabolism, pollutants, medicines, and/or dietary supplements), activate the AhR, and mediate downstream modulation of local inflammation through induction of diverse gene batteries within multiple intestinal cell types. 

IECs comprise the mucosal barrier and regulate critical physiological functions such as nutrient absorption, metabolism, secretion, permeability, and mucosal healing necessary to regenerate the intestinal epithelial barrier following injury. Following AhR activation, IECs maintain tight junctions necessary to limit leaky barriers and mucosal atrophy. Among intestinal immune cells, the AhR plays an important role in maintenance, generation, and differentiation of innate and adaptive immune subtypes. Intraepithelial lymphocytes (IELs) are responsible for gut mucosal barrier integrity and maintenance of these T cells depends on AhR activation. AhR-dependent regulation of anti-inflammatory signaling has been shown in IELs to confer protection from pathogenic insult and injury within the gut [18]. AhR controls the early differentiation of IL-17-producing helper T (T_h_17) cells, which play a key role in balancing physiological functions and pathophysiological pathways by secreting T_h_17 cytokines involved in autoimmune tissue inflammation and diseases following secretion of cytokines. Ligand-dependent activation of AhR can promote the differentiation of the immunosuppressive regulatory T cells (FoxP3^+^) [19], as well as type 1 regulatory T cell-like cells (IL-10^+^Tr1) [20], which dampens intestinal inflammation by producing IL-10 and CD-39; however, dysregulation of the AhR pathway can stimulate manifestation of autoimmune disorders such as experimental autoimmune encephalomyelitis [19], experimental autoimmune uveoretinitis [21], or spontaneous autoimmune diabetes [22]. AhR activation drives production of the key intestinal homeostatic inflammatory mediator, IL-22, by T_h_22 cells to regulate highly proliferative tissues involved in reproduction and health of IEC. AhR signaling controls differentiation of distinct T cell populations and functional activities of antigen-presenting cells by driving tolerogenic CD103 dendritic cells to maintain immune homeostasis within the gut. Overall, the AhR is highly expressed across epithelial and immune cells within the gut and is responsible for regulation of diverse inflammatory pathways.

## 3. Therapeutic Landscape of AhR-Targeted Molecules for Combatting Inflammatory GI Indications

**Figure 1:**
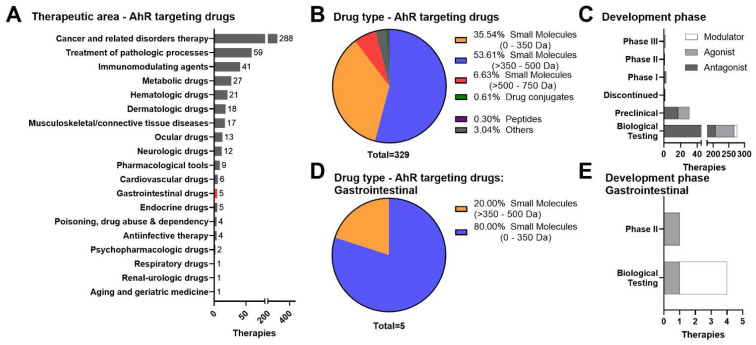
Meta-analysis of the current AhR therapeutic landscape. We searched for compounds targeting the AhR in humans using Cortellis Drug Discovery Intelligence and identified 329 drugs and biologics targeting AhR across all therapeutic areas. (**A**) The top therapeutic indications are cancer, general pathological processes, and immunomodulating drugs. (**B**,**C**) Of these, the 316 are small molecules, and the majority (276 compounds) are in early stages of biological testing and have not reached preclinical or clinical testing. (**A**,**D**) In this list, there are 5 small molecules intended to treat GI diseases. (**E**) The 4 molecules undergoing biological testing are indicated for IBD treatment.

High expression of the AhR within intestinal epithelial and immune cells coupled with its role as a prominent transcription factor involved in adaptive and innate immune signaling makes the AhR a desirable immuno-modulatory target for promotion of mucosal healing and maintenance of barrier integrity for patients. Utilizing Cortellis Drug Discovery Intelligence [23] to conduct a meta-analysis for new molecular entities (NMEs) targeting the AhR, we identified 329 NMEs with only four therapies reported under active development across biological testing and preclinical and clinical phases (Phases I–III) (Figure 1A,C). Of these NMEs, 316 are small molecules and 284 are in the early stages of biological testing (Figure 1B,C). When we filter our search to drugs/biologics that target the AhR specifically for GI disorders, the data reveals five therapeutics across biological and clinical phases (Figure 1D,E). Analysis of SAhRMs identified nine small molecules that modulated the AhR and three in development for Crohn’s disease (Figure 1C,E). Interestingly, investigation into new therapeutic modalities that may offer enhanced selectivity and serve as novel approaches for targeting the AhR for GI disease indications were not registered as NMEs under the GI category within Cortellis. To examine the landscape of emerging therapeutics targeting the AhR for inflammatory GI conditions, we reviewed the current progress of small molecules and new modality (e.g., microbial-derived, live biotherapeutic products, and probiotics, PROteolysis TArgeting Chimeras (PROTACs), and oligonucleotide) therapeutics within the field.

### 3.1. Small Molecule Approaches

**Figure 2:**
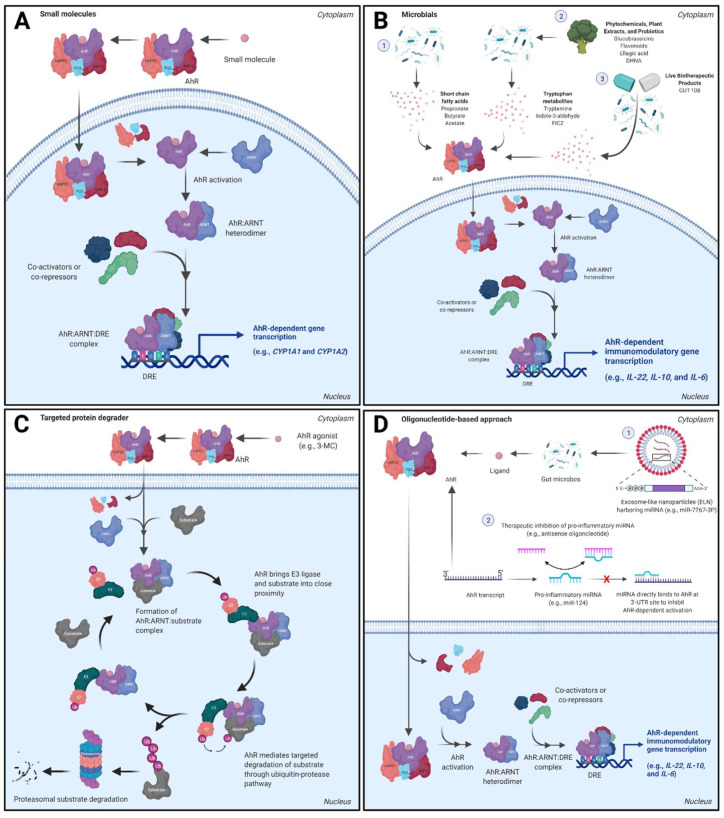
Therapeutic target modulation of the AhR for inflammatory GI conditions by chemical modality. (**A**) Small molecule approaches for systemic or GI-selective targeting of the AhR. The labile AhR exists within a multiprotein complex (protein 23 (p23), heat shock protein 90 (HSP90), and HBV X-associated protein-2 (XAP-2)) in the cytosol and upon ligand binding within the ligand binding domain (LBD), the AhR complex translocates into the nucleus. AhR activation occurs when the receptor dissociates from its chaperone proteins, heterodimerizes with ARNT, and selectively binds to DNA at the dioxin response element (DRE) site. This process is followed by recruitment of co-activators, co-repressors, and/or enhancers to the AhR:ARNT:DRE complex upstream of a promoter and induction of AhR-dependent gene transcription (e.g., *CYP1A1*). (**B**) Microbial therapeutic approaches for AhR modulation. Microbial AhR ligands include: (B1) gut microbiota-derived short chain fatty acids, (B2) plant diet- and microbiota-derived metabolites (e.g., Trp), and (B3) LBP microbiota-derived metabolites. Microbial AhR ligands can activate the AhR similar to small molecules and mediate AhR-dependent inflammatory gene transcription (e.g., *IL-22*). (**C**) AhR modulation by PROTACs. Apart from activating the canonical DRE-driven pathway, an AhR agonist can potentially also serve as a PROTAC by forming an E3 ligase:AhR:substrate ternary complex to promote proteasomal degradation of target protein of interest (substrate). (**D**) Oligonucleotide-based approaches for AhR target modulation. (D1) Exosome-like nanoparticles (ELN) harbor miRNA that stimulate production of gut microbiota-derived AhR ligands to activate the AhR and mediate intestinal homeostasis. (D2) Specific miRNA (e.g., *miR-124*) can promote intestinal inflammation by inhibiting the AhR; however, targeted inhibition or degradation of pro-inflammatory miRs can potentiate AhR-dependent gut resiliency.

The AhR is widely acknowledged to be a promiscuous xenosensor for structurally diverse small molecules and physicochemical properties influence AhR binding affinity and level of activation [24] (Figure 2A). This section will focus on the latest developments regarding clinical phase small molecule AhR ligands for therapeutic modulation of the AhR signaling pathway within the gut.

Multiple AhR agonists have been tested in inflammatory bowel disease clinical trials that were originally derived from traditional medicines. Structurally, each of these agents resemble indole-derived metabolites or contain polyaromatic groups. Indole and indirubin are potent AhR agonists found in traditional medicine known as Inidigo naturalais (IN) or qing-dai [25,26]. IN is an herbal extract from plants such as Indigofera tinctoria, Strobilanthes cusia O Kuntze, and Polygonum tinctorium Lour, and reports of IN use as an anti-inflammatory medicine date back to the 10th century. Contemporarily, IN is used in China as a treatment for ulcerative colitis, psoriasis, oral ulcers, radiation proctitis, chronic myelocytic leukemia and herpes zoster [25], and recently has been evaluated in multiple UC clinical trials [27,28]. Within an 8-week randomized placebo-controlled UC trial, IN (0.5–2.0 g oral per day) was effective at inducing a clinical response based on rates of mucosal healing defined as a Mayo endoscopic score of <1 and remission. Despite these promising results, long-term use may be limited by pulmonary arterial hypertension and liver dysfunction [27]. The oral small molecule, laquinomod, has also been evaluated as an immunomodulatory therapeutic for combatting Crohn’s disease. Treatment of CD patients within an 8-week clinical trial revealed that 0.5 mg laquinomod improved response and remission rate within exposed individuals [29]. While further development of laquinimod for IBD indications was halted, subsequent clinical testing was pursued for multiple sclerosis and Huntington’s disease. 

Difficulties relating to low activity, poor pharmacokinetic profile, post-translational modifications, and off-target effects have limited small molecule AhR therapeutic efficacy [30,31,32]. With the advent of AhR ligands within the clinic, a wave of rationally-designed AhR agonists for inflammatory GI indications within the preclinical space are being created and offer promising approaches to AhR target modulation with small molecules [14,33]. Pro-drugs or “pro-ligands” that serve as precursors to chemically transformed high affinity AhR ligands following microbial metabolism are in development for inflammatory GI disease indications that can mitigate metabolic stability issues observed with indole-containing AhR ligands, such as 6-formy-lindolog [3,2-b] carbazole (FICZ) and 2-(1′ H-indole-3′-carbonyl)-thiazole-4-carboxylic acid methyl ester (ITE) [14,34,35]. Further, design of small molecules with improved physicochemical properties and/or enhanced selectivity for the GI tract have also been investigated and offer a promising strategy for mitigating safety liabilities associated with systemic AhR activation [36]. Co-delivery of AhR agonists (i.e., laquinimod) with lipid nanoparticles has already been employed for neurological indications and offers the potential for targeted drug delivery to the gut for therapeutic modulation of the AhR [37,38].

### 3.2. Dietary Metabolites, Microbials, and Live Biotherapeutic Products

Several AhR ligands derived from the diet and/or gut microbiota metabolism have been identified to play a role in gut homeostasis and inflammation. Studies evaluating phytochemicals and plant extracts (e.g., flavonoids and urolithins, norisoboldine, indigo, IN, and glucobrassicins (broccoli extracts)), microbial-derived short chain fatty acids (e.g., acetate, propionate, and butyrate) [39], and Trp-derived microbial metabolites (e.g., tryptamine, indole-3-pyruvic acid, indole-3-acetaldehyde, indole lactic acid, indole-3-acetic acid, tryptophol, indole acrylic acid, indole propionic acid, FICZ, and others [40]) support AhR activation and either agonist, antagonist, or SAhRM activity by these compounds leading to direct or indirect mediation of anti-inflammatory pathways and overall gut homeostasis (Figure 2B). Commensal microbiota can influence AhR-mediated pro- or anti-inflammatory signaling following metabolism of endogenous ligands [41]. Detailed enzymatic generation of diverse dietary- and microbial-derived AhR ligands and potential roles in intestinal homeostatic signaling have been recently reviewed [42,43,44]. 

Gut dysbiosis and reduction in host- and microbial-derived AhR ligands can drive altered microbiota surveillance and immunoregulatory responses culminating in impaired mucosal immunity and increased severity in patients with IBD; however, several gut microbiota-derived metabolites have been identified to confer a protective role in gut immunity and offer potential therapeutic promise. Perhaps the most widely studied commensal microbiota-dependent metabolites are derived from the essential amino acid tryptophan, and collectively, Trp metabolites have been shown to play an AhR-dependent protective role in gut immunity. Fewer endogenous Trp metabolites are produced by intestinal microbiota under pathogenic conditions relative to those produced within homeostatic microenvironments. In fact, fecal microbiota analysis confirmed reduced AhR ligand production and AhR activation within stool from celiac patients compared to normal patients [45], and a negative correlation was observed between serum levels of Trp and disease activity within a cohort of IBD patients [46]. The inverse correlation of Trp metabolites and disease was also detected in serum and feces in dextran sulfate sodium (DSS)-induced colitis rats [47]. When the Trp metabolites, indole-3-ethanol, indole-3-pyruvate, and indole-3-aldehyde, were pre-administered to DSS-induced mice, it was found that effects of each metabolite were partially dependent on AhR activation [48]. Apart from Trp metabolites, urolithins (UroA and UroB) result from catabolism of dietary polyphenols in a multi-step reaction. As a selective human AhR antagonist, UroA has been shown to attenuate inflammation and maintain tight junction formation within Caco-2 cells following inhibition of IL-6 and PTGS2 transcription in an AhR-specific manner [45,49]. Plant-derived flavonoids, such as alpinetin, have demonstrated efficacy against colitis following direct AhR activation and immunomodulation via promotion of Treg differentiation [46,50,51]. Lastly, short chain fatty acids illustrate anti-inflammatory activity, enhanced barrier integrity, pathogen protection, and a role in resistance to colon tumorigenesis via either direct AhR activation or indirectly by upregulating expression AhR-dependent IL-22 production [52,53]. 

In contrast to dietary- and gut microbe-derived AhR ligands that mediate intestinal homeostasis, oxazalone has been shown to induce AhR-dependent colitis following downregulation of anti-inflammatory factors (i.e., Il-10) in mouse models [54]. Further, IN demonstrated amelioration of colitis within the DSS mouse model, but exacerbated colitis in an oxazolone-induced colitis model via alterations in gut microbial composition [55]. Notably, gut microbiota-derived metabolites have been shown to modulate systemic inflammatory responses through the gut–liver [56], gut–brain [57], gut–skin [58], gut–lung [59], and/or gut–pancreas [60] axes. Gut microbiota-derived AhR ligands also synergistically enhance basal and ligand-induced and AhR-dependent CYP1A1 and CYP1B1 expression within intestinal cells [61]. Thus, the diversity of dietary- and gut microbe-derived AhR ligands requires careful attention to potential drug–drug interactions, pathogenic environment, and diet which could influence microbiota composition and whether an AhR ligand protects or exacerbates AhR-dependent regulation of inflammatory pathways both within and beyond the GI tract.

Treatment with AhR activators is an attractive therapeutic strategy in GI inflammatory conditions. Fecal microbiota transplantation (FMT) is one therapeutic approach that relies on large amounts of intestinal microbiota transferred from prescreened healthy donors to the GI tract of recipients to help correct a dysbiosis condition. FMT alleviates DSS-induced colitis in mice through increasing the secretion of anti-inflammatory cytokines by activating AhR signaling [62]. Alternatively, use of live microorganisms represents an intriguing therapeutic approach for amelioration of IBD while overcoming some of the disadvantages associated with FMT (e.g., variable efficacy and undesirable safety risks from different stool samples). 1,4-dihydroxy-2-naphthoic acid (DHNA) is a probiotic AhR activator that has been shown to induce antimicrobial protein production, alter microbiota composition, and inhibit DSS-induced colitis in mice [63]. Treatment with the 8-strain bacterial probiotic, VSL#3, effectively induced remission in ulcerative colitis patients [64,65]. In mice, *Lactobacillus reuteri* can metabolize Trp to indole-3-aldehyde and can drive AhR-mediated transcription of Il-22 leading to balanced mucosal response [66]. Live biotherapeutic products (LBPs) are a newer therapeutic modality providing an alternative to anti-inflammatory or immunosuppressive agents. A recent publication demonstrated that the rationally-designed 11-strain LBP consortium, GUT-108, had strains representing multiple phylum that synthesized the Trp metabolite indole. In an experimentally-induced colitis model using germ free Il-10^−/−^ mice, GUT-108 colonization helped correct dysbiosis via modulation of AhR pathway genes [67]. Further, three dietary supplements that activate the AhR have been launched to date, including diosmin, diosmin/hesperidin, and benvitomod [23].

For microbial, probiotic, and LBP safety assessment, correct strain identification is critical to determine the AhR activation potential. Additional safety components that have been assessed in a de-risking strategy include screening for genetic factors that predispose patients to increased disease severity (e.g., caspase recruitment domain-containing protein 9 (CARD9) polymorphism [68,69,70]), whole genome sequencing, the presence of antibiotic resistance genes, virulent-related genes, mucin degradation ability, and the formation of biogenic amines and other harmful metabolic enzymes. For probiotics, single and repeat dose animal toxicity studies have been conducted to address safety questions specific to the strains being tested [71]. Similar to probiotics, strain identification and characterization is essential to an LBP safety assessment [72]. While commensal bacteria are capable of biotransformation and generation of AhR ligands with beneficial effects, the gut microbiome can alter the disposition and toxicity of drugs in detrimental ways. For example, microbiota obtained from human colon digests were shown to be capable of biotransforming AhR agonists naphthalene, phenanthrene, pyrene, and benzo(a)pyrene in one study. The metabolic reaction products, unlike the parent compounds, exhibited estrogenic activities [73]. As microbials, probiotics, and LBPs may be consumed concomitantly with other drugs that are affected by the same enzymes, future safety screens should assess drug metabolizing enzymes. Enhanced understanding of the drug metabolizing enzymes (cytochrome (CYP) P450 gene expression and phase II enzymes) and microbiota landscape across diseased and normal patients will aid in elucidating potential AhR ligands and/or metabolites formed following exposure to microbial, LBP, probiotic, and/or dietary supplements, which can facilitate early methodologies surrounding de-risking strategies. Further, novel systems biology approaches for gut microbiota–host interactions can aid in understanding crosstalk between organ systems as well as distinct intestinal subtypes (e.g., immune versus epithelial cells) [74]. The quantification of microbial-derived metabolites in preclinical animal or clinical fecal contents could also provide insight into species-specific, wild-type versus germ-free or diseased animals, and normal compared to diseased patient AhR ligand landscape, which is critical for understanding the potential for sustained AhR activation [75].

### 3.3. AhR Proteolysis Targeting Chimeras (PROTACs)

Two decades ago, Ray Deshaies, Crews, and colleagues [76] demonstrated PROTACs can be successfully used to target proteins for degradation. Ever since, the targeted protein degradation (TPD) field has demonstrated significant utility to engage targets previously thought to be undruggable. This has opened multiple avenues to help patients with unmet medical needs. PROTACs exploit the well-established ubiquitin–proteasome system (UPS) as a primary mechanism for proteasomal degradation [77]. In essence, a PROTAC is a heterobifunctional molecule that recruits a target protein of interest to a specific E3 ligase complex for ubiquitination and subsequent degradation via 26S proteosome. The detailed UPS system and utility of PROTACs are reviewed elsewhere [78,79]. As an alternative to the classical small molecule inhibitors which tend to impede protein function via occupancy-driven mechanism, the event-driven goal of PROTACs is to eliminate target protein via degradation and thereby providing a novel molecular probe to interrogate target protein function in a complex signaling pathway. Despite their existence over the past two decades, true utility of PROTACs has started to emerge over the past few years. Currently, few PROTACs have made it into clinical trials with the majority of them targeting nuclear receptors for degradation [80,81].

A possibility of AhR-PROTAC was originally hypothesized by Kim, Swanson, and colleagues [82,83]. They identified apigenin as a partial AhR antagonist that can directly interact with AhR and inhibit its dioxin response element (DRE)-driven transcriptional activity. They synthesized an apigenin-based PROTAC (Api–PROTAC) as a molecular probe to elucidate AhR biology. The authors found that modifications carried out on any of the free hydroxyl groups on apigenin to recruit an E3 ligase recognition residue, maintained its ability to inhibit AhR-induced transcriptional activation. This allowed successful synthesis of Api-PROTAC by connecting apigenin to Von Hippel-Lindau (VHL) E3 ubiquitin ligase recognition motif via linker at 4′-hydroxyl group on apigenin. The designed Api-PROTAC was effective in degrading AhR in mouse hepatocyte cells. Addition of the proteosome inhibitor, epoxomicin, abolished Api-PROTAC-mediated AhR degradation suggesting the degradation was proteosome-dependent [83]. In a follow-up study, the authors continued to demonstrate Api-PROTACs can degrade AhR in human cells in the presence of prototypical AhR ligand, TCDD. It also inhibited TCDD-induced CYP1A1 protein levels in NHK cells [82]. It was further confirmed by the authors that Api-PROTAC inhibits the ability of TCDD to induce AhR:ARNT binding to dioxin-response elements and hence limiting TCDD-mediated induction of CYP1A1 and CYP1B1 mRNA expression [82]. While API-PROTAC is an exciting molecular probe, degradation of AhR might serve deleterious in the context of IBDs, where activation of the AhR signaling pathway is considered protective.

Another groundbreaking work in AhR biology was led by Kato and colleagues [84] who demonstrated the role of activated AhR as an atypical component of E3 ubiquitin ligase complex in degradation of sex hormone receptors. It is previously known that TCDD can modulate transcriptional activity of estrogen receptor-α (ER-α), self-ubiquitinate, and degrade via cullin 4B (CUL4B)-dependent and -independent pathways [85,86,87,88]. The authors demonstrated that 3-methylcholanthrene (3-MC)-activated AhR can form a CUL4B^AhR^ ubiquitin complex made-up of CUL4B, damaged-DNA-binding protein 1 (DDB1), ARNT and transducin-b-like 3 (TBL3) together with RBX1 (ROC1). Ligand-dependent activation of AhR was required to act as a substrate-specific adaptor component of the CUL4B^AhR^ complex to promote ER-α and androgen receptor (AR) degradation via 26S proteosome. Since degradation of a substrate is an event-driven phenomenon, a ligand bound AhR:E3 ligase complex may continue to recruit new substrates until the AhR agonist is metabolized or can no longer maintain an active AhR:ligase complex (Figure 2C). The ubiquitin ligase function of AhR was independent of its transactivation function as demonstrated by Poellinger and colleagues [89]. AhR partial agonist/antagonist α-naphthoflavone (α-NF) failed to enhance ER-α degradation, suggesting only AhR agonists can cause this confirmational change [85]. Taking inspiration from Kato and colleagues, Ohoka and colleagues [90] developed two small molecule chimeras using β-NF to recruit atypical AhR:E3 ligase complex to degrade cellular retinoic acid binding proteins (CRABP-1 and 2) and bromodomain containing (BRD) proteins via UPS further confirming the original hypothesis. 

Overall, these findings are exciting in terms of therapeutic utility of AhR as a target of interest for proteasomal degradation in inflammatory diseases or certain cancers where endogenous overactivation of the AhR is consistently reported [91,92,93]. However, it makes more sense from an IBD perspective wherein one can exploit this unique AhR:E3 ligase complex phenomenon by synthesizing heterobifunctional AhR:E3 ligase chimeras wherein the AhR:E3 ligase can facilitate degradation of a target protein(s) of interest in IBD. Further, similar to the molecular glue concept already established for cereblon immunomodulatory agents [94], one can plausibly hypothesize molecular glue-like activity where a ligand-activated AhR:E3 ligase complex in the gut can degrade currently unknown and undruggable targets implicated in IBD. While TPD is an exciting modality, the success of it lies in how we balance the unknown safety risks associated with AhR activation and AhR:E3 complex mediated protein degradation. Apart from substrate degradation-dependent exaggerated pharmacology, unknown neo-substrate degradation is of toxicological concern with AhR:E3 ligase-mediated TPD. As of this review, no AhR degraders (AhR-PROTACs) or AhR agonists that utilize this AhR:E3 ligase phenomenon for TPD are being pursued to illustrate the toxicologic limitations of this modality.

### 3.4. Oligonucleotide-Based Approaches

Oligonucleotide therapeutics encompasses antisense oligonucleotides (ASO), small-interfering RNAs (siRNA), microRNA (miRNA), and aptamers. At the time of this review, there are 10 approved therapies across the United States, Europe, and Japan and their target tissues include the central nervous system, muscle, liver, eye, and immune cells [95]. This class of drugs represents a promising alternative approach for treatment of GI-related disorders. 

Recent efforts have focused on the therapeutic development of ASOs and siRNA which can alter expression of target genes involved in the progression and disease development of IBD [96]. Primary mechanisms utilized in IBD-based therapeutics include the inhibition of mRNA translation and RNA interference (RNAi) [97]. Target molecules that have been explored in preclinical and clinical studies include TNF-α, ICAM-1, TLR-9, SMAD7, CHST15, GATA3, and RELA [98,99]. However, clinical trials with these oligonucleotide therapies were disappointing due to a lack of efficacy [97,100,101]. For example, mongersen (GED-0301), an ASO targeting the intracellular protein SMAD7, which inhibits TGFβ1/SMAD signaling, showed great promise in phase I and II clinical trials for Crohn’s disease patients [102]. Although the subsequent phase III trials were terminated due to lack of efficacy, the previously collected clinical and endoscopic data are still encouraging for the application of ASO therapeutics for IBD treatment. The lack of translation of promising preclinical oligonucleotide findings into clinical trials could be attributed to several factors, including target selection and delivery methods. The delivery of oligonucleotide therapeutics to target tissues and organs such as the colon can be challenging. In order to overcome this limitation, new strategies for oral delivery of ASOs such as polysaccharide-based nanocomposites and microspheres with colon-specific design for treating IBD are being explored [103,104]. 

Identification of molecular targets can be difficult due to the number of genes involved in the gut inflammation response. One immunological target that has yet to be evaluated as an efficacious treatment option is the AhR. Based on the role of AhR in intestinal homeostasis, it is plausible that the AhR or AhR signaling pathway may be a potential target for GI disease treatment. Further, expanding ASO target molecules to include the AhR would take advantage of the ability of ASO to achieve selectivity and to regulate transcriptionally instead of through ligand activation. MiRNA offer an alternative therapeutic target in IBD due to their involvement in epithelial barrier disruption and dysregulation of the immune system. Target modulation of miRNA precursors also represents a novel approach for various inflammatory GI conditions [105]. *MiR-124* has been implicated in induction of intestinal inflammation through the inhibition of the AhR; therefore, initiating key events in the pathogenesis of Crohn’s disease [106] (Figure 2D). In addition, tetrandrine, a plant-derived natural agonist of AhR, downregulated *miR-429* expression in a colitis mouse model. This resulted in an upregulation of Occludin expression, a key transmembrane protein, consequently mitigating intestinal epithelial barrier defects in an AhR-dependent manner [107]. The AhR-miR-212/132 axis has also been shown to promote intestinal inflammation within DSS-induced colitis mice via induction of T_h_17 cells and downregulation of Il-10-producing T cells [108]. In contrast, several miRs (e.g., *miR-590-5p*, *miR-19b*, and *miR-876-5p*) have been identified that promote intestinal homeostasis and modulation of these precursors offers a novel approach to reduce intestinal inflammation [109,110,111]. Ginger and broccoli exosome-like nanoparticles (ELN) have also been developed, which harbor plant-derived exosomal microRNA (e.g., *miR-7267-3P*) that alter microbial metabolism of AhR ligands (e.g., indole-3-aldehyde) and drive downstream Il-22 production and amelioration of colitis [112,113] (Figure 2D). Although preclinical and clinical trials have highlighted the potential of oligonucleotide-based therapies for treating patients with GI diseases, additional research is needed to develop effective therapeutic strategies for patients.

## 4. Addressing AhR Safety Liabilities Going Forward

Decades of research evaluating dioxin-mediated AhR activation and constitutively activated AhR transgenic models has provided a robust dataset of AhR-dependent toxicities in vivo. Within rodent models, sustained AhR activation has resulted in an array of organ-specific toxicities, including thymic, bone, immune, circulatory, cardiovascular, metabolic, hepatic, GI, skin, respiratory, lymphatic and hematopoietic, reproductive, and developmental system effects [114,115,116,117,118]. AhR agonists have demonstrated tumorigenicity across a wide variety of organ systems via alterations to DNA, changes in expression of genes relevant to carcinogenesis, inhibition of apoptosis, suppression of intercellular communication, stimulation of proliferation of preneoplastic stem cells, and indirect signaling in an AhR-specific manner [119]. Apart from systemic approaches to target the AhR, GI-selective modulation poses safety concerns, including enhanced susceptibility to infection and reduced bacterial infection clearance [120], impaired mucosal immunity [121,122], antibiotic resistance [123], attenuation or exacerbation of inflammation [124], and increased cancer risk from generation of procarcinogens [93,125]. Notably, the potential for AhR ligands and microbiota-derived metabolites to permeate through leaky barriers within inflammatory GI disease states can also result in systemic exposure concerns.

Epidemiological cohorts exposed to dioxins have also been studied to understand sustained AhR-dependent activation and elevated cancer incidence, morbidity, and/or mortality within exposed populations, producing variable results. Lung, lymphatic and hematopoietic, soft tissue sarcoma, GI, rectal, breast cancer, multiple myeloma, biliary tract, and vaginal cancer were reported in one or more of the cohorts [126]. Further, noncancer endpoints were also noted including chloracne, diabetes and increased serum triglyceride levels, altered thyroid function, cardiovascular disease, increased immunoglobulins and complement proteins, reproductive effects, and developmental effects [126]. While single nucleotide polymorphisms (SNPs) have been identified in humans, no AhR SNPs have been significantly associated with disease incidence; however, some SNPs have been suggested to result in varied gene expression patterns and downstream signaling [127,128]. Genome-wide association studies have also identified SNPs within genes directly associated with the genomic AhR signaling pathway that might impact AhR binding sites, AhR target gene expression, and inter-individual variability in AhR-dependent toxicity [129,130,131,132].

### 4.1. Confirmation of AhR Canonical Signal Transduction as an Early De-Risking Strategy

**Figure 3:**
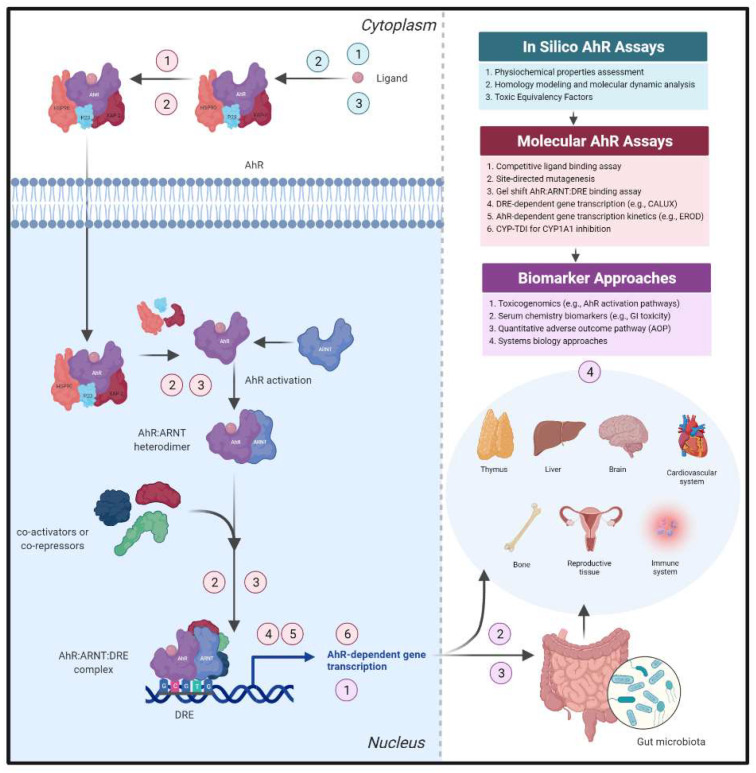
Preclinical safety approaches for de-risking sustained AhR activation. AhR activation has been demonstrated to drive AhR-dependent toxicological outcomes; therefore, development of de-risking approaches for sustained AhR activation could aid in compound prioritization. In silico, molecular assay, and biomarker approaches can be utilized within a tiered screening approach to elucidate AhR-dependent mechanism of action for hazard identification and potential risk within and beyond the GI tract.

The labile AhR exists within a multiprotein complex (protein 23 (p23), heat shock protein 90 (HSP90), and HBV X-associated protein-2 (XAP-2)) [120] in the cytosol and upon ligand binding within the ligand binding domain (LBD), the AhR complex translocates into the nucleus. AhR activation occurs when the receptor dissociates from its chaperone proteins, heterodimerizes with ARNT, and selectively binds to DNA at the DRE site [133]. This process is followed by recruitment of co-activators, co-repressors, and/or enhancers [134] to the AhR:ARNT:DRE complex upstream of a promoter and induction of AhR-dependent gene transcription [24,135,136] (Figure 3). Decades of research support the concept of DLCs serving as tumor promoters through sustained AhR activation and potential for AhR ligands to be biotransformed by CYP1A1/1A2 to carcinogens; however, the role of the AhR in biological and physiological functions is less understood [137]. 

Early de-risking strategies should focus on delineating whether a therapeutic compound activates the AhR through the canonical pathway as a first step in hazard identification (Figure 3). Measurement of ligand binding within the AhR LBD using a competitive radiolabel AhR ligand binding assay is required to definitively categorize a compound as an AhR ligand [138,139]. Additional techniques harnessing non-radiolabel receptor binding approaches have also been recently developed [140]. An understanding of whether an AhR ligand and potential downstream metabolite(s) act as agonists, antagonists, and/or SAhRMs can inform what specific AhR-dependent pathways or cellular crosstalk partners may be involved, especially for microbial metabolites where little is known regarding how dose–response and metabolic activity alters intestinal microenvironment interactions. In vitro gel shift analysis can determine whether a ligand:AhR:ARNT:DRE complex is formed [141], selectively binds to a specific DRE nucleotide sequence [142,143], and confirm AhR-dependent gene expression resulting from a ligand-activated complex directly binding to DNA in an AhR:ARNT:DRE specific manner. Structurally diverse ligands differentially interact with amino acid residues within the AhR LBD to generate specific conformational changes that can result in diverse ligand-dependent AhR pathways, molecular crosstalk, and recruitment partners, and ultimately transcription of a constellation of genes that mediate various signaling cascades [144,145,146]. Homology models of the AhR ligand binding pocket across various species [147] as well as a structural model of the AhR:ARNT dimer that encompasses the entire bHLH-PASA-PASB domain regions [148,149] have been generated and can aid in predicting new AhR ligands and allow comparison of structurally diverse ligand binding conformations to elucidate mechanisms of ligand- and AhR-dependent toxicity and biology [150,151,152]. Further, recent generation of an AhR:ARNT:DRE crystal structure has been reported, which offers insight into potential dynamic structural hierarchy of the activated AhR [153]. 

Many small molecules and candidate endogenous ligands contain indole structures that have demonstrated strong species specificity for the human AhR relative to murine AhRs [154,155,156]. Ligand-selective and cross species comparisons of the molecular AhR pathway can be further investigated through various in vitro assays that incorporate species-specific AhR, ARNT, point mutations, and cellular microenvironments to assess relative levels of ligand binding, AhR activation, and AhR-dependent gene expression across ligands and/or species for hazard identification in early discovery toxicology programs [141,154,157]. Reporter-based assays, such as the chemically activated luciferase expression (CALUX) bioassay [158], that harbor DRE(s) upstream of an AhR-dependent gene promoter (i.e., CYP1A1) and generate relative light unit output as a measurement of ligand-, AhR-, and DRE-dependent luciferase gene transcription or the widely utilized ethoxyresorufin-O-deethylase (EROD) assay [159] can be used for high-throughput screening of compounds for AhR activation kinetics across species and potentially across modalities. Examination of the ability of an AhR antagonist (e.g., CH-223191) to inhibit compound-dependent *CYP1A1* induction can identify a novel AhR agonist [160], and evaluation of whether a given AhR ligand and downstream metabolite(s) inhibit CYP1A1 (e.g., CYP time-dependent inhibition) informs potential alterations in drug clearance and oscillatory AhR activation [161]. Ultimately, it is critical to consider not only potency and efficacy, but kinetic properties (e.g., uptake, distribution to target organs, metabolism, and clearance) within in vitro and in vivo studies to effectively assess potential safety liabilities from small molecule AhR modulators. In addition, mechanistic studies focused on de-risking prominent AhR-dependent toxicities early in a program can incorporate ad-hoc assays into in vitro tiered screening approaches, such as evaluation of AhR ligand-dependent inhibition of estrogen-activated reporter gene activity from a consensus estrogen response element (ERE) as an early safety indication for AhR-dependent endocrine disruption [162]. Prioritizing compounds based on *CYP1A1/1A2* induction levels, metabolite profile, along with assessment of cell proliferation biomarkers, can also aid to de-risk potential carcinogenicity liabilities and develop improved SAR within the early discovery toxicology space [163]. Utilizing these molecular approaches in a safety-by-design strategy can support development and trigger early de-risking strategies for novel therapeutics targeting the AhR.

### 4.2. Harnessing AhR-Dependent Transcriptomic Profiles to Identify Safety Thresholds

Sustained AhR activation by DLCs and subsequent AhR-dependent gene expression is associated with diverse organ- and tissue-specific toxicities; however, continuous AhR activation by nongenotoxic compounds and/or transient/oscillatory activators, such as Trp metabolites, can also result in activation of AhR-dependent gene expression batteries that will potentiate target organ toxicities. Steps have been taken to establish thresholds for AhR-dependent toxicity based on known physicochemical properties and structure (i.e., Toxic Equivalency Factors (TEFs)), which has aided in assessing AhR activation associated with dioxin-like toxicity as an approach for risk assessment; however, this system fails to predict interspecies sensitivities and is not applicable to structurally diverse ligands [1]. Whether small molecule drug candidates result in dietary, environmental, or microbial metabolism that drives prolonged gene expression patterns following AhR therapeutic modulation, remains to be seen. 

Despite these concerns, efforts to better understand AhR-dependent mechanisms underlying toxicological responses and individual susceptibility have progressed using various omics approaches (e.g., genomics, epigenomics, proteomics, metabolomics, and transcriptomics). Toxicogenomic profiling offers a high-throughput methodology to extract mode of action (MOA) information from complex RNA expression datasets, which can enable transcriptomic signatures of ligand- and AhR-dependent toxicity. To date, most transcriptomic biomarker research within the field of toxicology has focused on liver carcinogenesis; however, many of these efforts incorporate AhR activation as a key biomarker for drug-induced carcinogenicity and a combination of AhR activation with additional transcriptomic signatures can aid in development of quantitative effect thresholds associated with AhR-dependent GI toxicological events [164,165]. Identification of a transcriptomic signature for in vivo rodent carcinogenicity based on prominent genes involved in molecular initiating and/or key events (e.g., AhR activation) from quantitative adverse outcome pathway (AOP) analysis revealed >90% accuracy of gene expression biomarkers for predicting rodent carcinogenesis within a 134-compound validation set [166]. Similarly, Hill et al. [165] identified molecular tipping points for liver carcinogenicity based on genomic biomarkers spanning genotoxicity, cytotoxicity, and activation of transcription factors (AhR, CAR, ER, and PPARα) that demonstrated 97% accuracy for identifying carcinogens within the Toxicogenomics Project-Genomics Assisted Toxicity Evaluation System (TG-GATES) [167] training set (77 compounds). Effectively, Qin et al. proposed the development of biologically relevant thresholds for AhR-dependent toxicological action based on key transcriptomic biomarkers (*Cyp1a1* and *Cyp1a2*) within 4-day rodent studies. Thresholds for carcinogen and non-carcinogen AhR activation were selected based on weighted average of Log_10_ *Cyp1a1* and *Cyp1a2* induction at different time points. This approach enabled categorization of compounds as high or low concern for AhR-dependent carcinogenicity and demonstrated low concern compounds lacked sustained AhR activation with continued dosing [168]. Taylor and colleagues [163] reported utilization of an AhR gene panel (*Cyp1a1*, *Cyp1a2*, NAD(P)H quinone oxidoreductase (*Nqo1*), and epoxide hydrolase (*Ephx*)) and implementation of threshold gene expression values within rat studies to assess levels of AhR activation for hazard identification within a GlaxoSmithKline S1P1 program. In fact, Glaab et al. [169] reported 80–90% sensitivity and 100% specificity of gene expression signatures for predicting compound-induced liver, kidney, or smooth muscle tissue injury (i.e., degradation and necrosis) relative to serum clinical pathology markers (e.g., liver function enzymes) with 80–90% sensitivity and 100% specificity. Additional transcriptomic signatures that incorporate AhR activation have been validated to predict cellular responses associated with drug-induced and reactive metabolite-driven liver injury [170,171], and shown to differentiate genotoxic from non-genotoxic agents [172,173,174,175] and human-relevant from non-human-relevant MOA [176] within in vitro and in vivo studies. Limitations regarding diversity in GI toxicity phenotypes and functionality along induced focal injury within a certain location of the GI tract (e.g., jejunum, duodenum, ileum, or colon) has resulted in lack of robust biomarkers of GI injury; however, recent evaluation of a 12-transcript optimized algorithm demonstrated 68% sensitivity and 96% specificity for duodenal tissue degeneration/necrosis [169], which supports further adoption of toxicogenomics-based approaches to expand the pool of predictive GI biomarkers in conjunction with AhR pathways. Collaborative efforts across toxicology and GI drug discovery can aid in further validation of non-invasive, highly specific, and subtype-selective toxicogenomic biomarkers of GI injury. 

In this light, identification of compounds as transient or sustained AhR activators through transcriptomic approaches can serve as a strategy to flag potential AhR-dependent toxicity liabilities (Figure 3). Advances in toxicogenomic storage and analytical and reporting standards highlight the utility of this approach for robust and reproducible analyses [177]. Less developed AhR-dependent toxicological pathways (e.g., AhR inhibition and intestinal barrier permeability) can incorporate transcriptomic data and computational tools to support quantitative AOP development, definition of dose–response relationships, and designation of gene expression thresholds at which transcriptional alterations result in specific key events [178,179,180]. Predictive toxicogenomic strategies have the potential to (i) identify human-relevant on- and off-target effects, (ii) validate predictive biomarkers with key associative events for systems biology approaches, and (iii) bridge gaps between genotypic and phenotypic (e.g., histopathology) data to support decision-making [181].

### 4.3. Assessment of AhR-Dependent Pathways within Intestinal Microenvironment Cultures

Several intestinal in vitro platforms have been developed that can serve as predictive early screens for AhR target modulation within drug discovery programs. Classically, human immortalized colon cell lines (i.e., Caco-2 or T84) have served as high-throughput models for evaluating GI toxicity and drug absorption, distribution, metabolism, and clearance. Inherent limitations regarding lack of cell–cell communication and cell–environment interaction have resulted in poor translatability of the monoculture cell line models; although, addition of extracellular matrix, three-dimensional culture conditions, and intestinal epithelial or immune cell subtypes has improved predictivity. New methodologies that incorporate diverse IEC subtypes (i.e., Paneth, tuft, goblet, and enteroendocrine cells) in 3D microenvironments (e.g., colonoids, enteroids, and 3D microtissues) have demonstrated superior clinical translatability over cell lines and in vivo-like predictivity for GI toxicity [182,183]. In fact, AhR-deficient mouse organoids have been utilized to explore critical roles of the AhR within IECs [11]. Considering organoids can be biobanked for higher-throughput analysis, this offers a potential screening platform to explore AhR-dependent effects within a physiologically-relevant system. Further, the combination of 3D intestinal culture systems with microphysiological [184] systems and/or Transwell technologies has enabled elucidation of diverse mechanistic pathways within GI drug discovery. 

Utilization of advanced GI in vitro model systems that harbor microenvironment cellular and environmental crosstalk cues can provide a more physiologically-relevant system for assessment of AhR-dependent GI toxicity liabilities for hazard identification. GI inflammatory conditions can be triggered by microbial, pathogenic, or chemical stimuli and result in a sustained inflammatory response characterized by increased secretion of pro-inflammatory milieu and disruption of intestinal homeostasis. A number of cytokines/chemokines regulated directly (e.g., DRE sequence upstream of gene promoter) or by crosstalk through the AhR including, IL-6, IL-4, IL-17, IL-23, IFNγ, CCL20, CXCL5, IL-1β, IL-33, IL-10, and IL-27 could be evaluated as secreted predictive biomarkers of AhR-dependent immunomodulation in vitro and in vivo. Use of in vitro or ex vivo models to study host–microbiome interactions with normal and/or diseased-patient strains have shown promise in addressing microbiome signaling influences on intestinal pathogenesis as well [185]. Incorporation of new approach methodologies into early drug discovery will also aid in advancing mechanistic understanding of underlying AhR-dependent mechanisms of action and progress implementation of alternatives to animal use (i.e., 3Rs) within the field of inflammatory GI research.

### 4.4. Safety Assessment Concerns Regarding AhR Target Modulation

**Figure 4:**
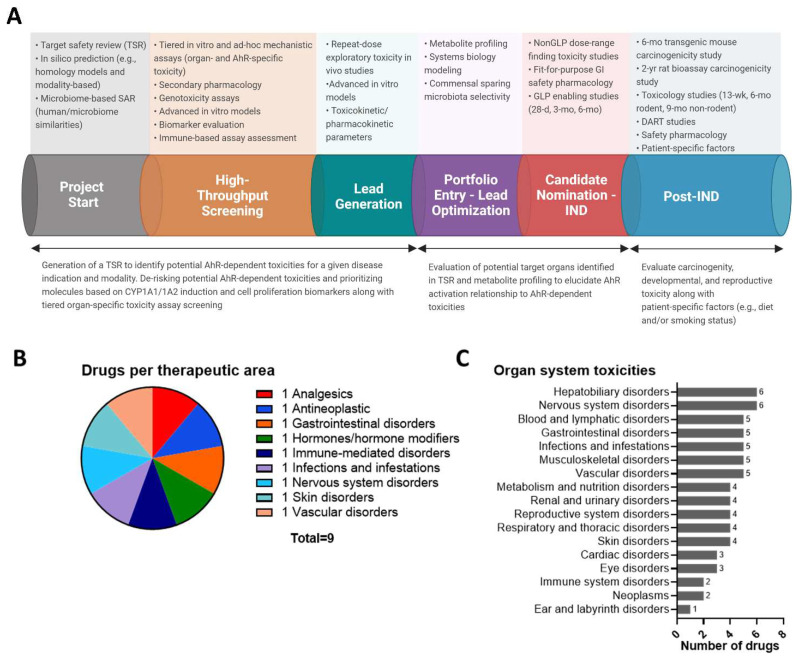
Roadmap for addressing potential AhR-dependent toxicity liabilities. (**A**) Roadmap for de-risking potential AhR-dependent safety liabilities throughout drug development. (**B**) Approved drugs that were identified to be AhR agonists [187] and were classified by therapeutic area according to FDA label or clinicalTrials.gov for non-approved drugs, laquinimod, and benvitimod. (**C**) Confirmed toxicities of the 9 therapeutics reported by regulatory agencies (FDA, EMA, HAS–SG, and TGA–AU) were obtained using OFF-X (https://targetsafety.info/: accessed on 22 February 2022) and grouped by the organ system.

To address on- and off-target safety liabilities pertaining to AhR modulation, a thorough review of potential target safety risks needs to be carried out (Figure 4A). The AhR is fairly conserved across species; however, significant differences in species, strain, and sex can result in altered levels of AhR activation, and determining which animal will be the most sensitive model for preclinical toxicity studies can depend on the chemical modality, target tissue, organ, cell type, underlying MOA, and physicochemical properties of a given AhR ligand [186,187,188,189]. Species-specific differences in ligand binding specificity, potency, and gene batteries have also been reported between rodent models and humans with limited translatability between in vitro and in vivo studies, which further complicates predictivity [155,190,191]. Apart from the AhR, associated proteins within the AhR signaling pathway (i.e., CYP1A1) also harbor cross species differences that need to be taken into account for predicting human toxicity. For programs with known lack of translatability between animal models and humans (e.g., oligonucleotides), development of cross reactive sequences and/or thorough mechanistic understanding of isoforms within surrogate species is necessary to assess translatability of toxicity in vivo. With low homology, likely one species will be identified to characterize on-target toxicity, while another species will be utilized for off-target assessment. Further, transgenic animals with humanized cells or tissues could help address cross species differences within toxicity studies [192]. Determination of desired AhR ligand affinity (high, medium, or low), potency, selectivity, and intrinsic activity (i.e., agonist, antagonist, and/or SAhRM) depends on disease indication, patient population, delivery methodology, targeted intestinal subtype, localization within the GI tract, and other drug development considerations. Incorporation of promiscuity screening panels can aid in early identification and de-risking of potential off-target hits and crosstalk mechanisms that may impact safety [193]. Identifying differences in mechanism and susceptibility through a comprehensive target safety review (TSR), conducting tiered in vitro screening with cells or sequences from preclinical species, and prioritizing clean compounds through hazard identification will help identify relevant model systems for safety pharmacology and toxicology studies as well as de-risk bad actors early within a drug discovery program.

Performing a short (3–14 day) repeat-dose exploratory in vivo study can aid in understanding toxicity liabilities early through the collection of AhR target organs of toxicity for histological analysis, clinical pathology (e.g., hematology, coagulation profile, urinalysis, and serum chemistry), evaluation of biomarkers of toxicity, and/or immunophenotyping, and clinical observations (e.g., organ and body weight). *Cyp1a1* can be utilized as a pharmacodynamic biomarker for AhR activation across target tissues to assess biodistribution and understand potential AhR-dependent safety liabilities with candidate molecules [14]. Utilization of exposure multiples that achieve at least 30-times the pharmacological dose and study design that incorporates conservative dosing regimens (based on literature review or preclinical studies) will ensure a robust toxicology study is carried out. Safety pharmacology studies can also provide information regarding adverse pharmacodynamic and/or pathophysiological effects relevant to human safety across organ systems.

Pharmacokinetic information (e.g., absorption, distribution, metabolism, and excretion) within in vitro and in vivo studies can provide relevant information pertaining to potential drug interactions and direct chemical SAR changes for improved target organ biodistribution (e.g., GI selectivity) and clearance within normal and diseased (e.g., DSS-induced colitis mouse) preclinical models. Chen and colleagues [14] utilized an in silico, in vitro, and in vivo hit-to-lead selection cascade to identify potent AhR ligands within an indole-containing structural library that yielded lead compounds with optimal pharmacokinetic parameters to achieve favorable oral bioavailability, potent nanomolar activity, and appropriate clearance required to limit compound accumulation and persistent AhR activation. Metabolic analysis is essential for predicting stability, reactive metabolite generation and downstream carcinogenicity risk through sustained AhR activation (e.g., autoinduction and/or frequent/chronic dosing). Limited metabolic stability coupled with low absorption can limit AhR ligand therapeutic efficacy in the liver, yet still maintain intrinsic activity and potency within the GI tract [36]. Design of pro-drugs with these pharmacokinetic parameters in mind have the potential for enhanced GI selectivity, while limiting systemic AhR modulation. Incorporation of early in vitro assays to assess reactive metabolite generation through glutathione (GSH) consumption and electrophilic or nucleophilic trapping (e.g., GSH or potassium cyanide (KCN) trapping) [194,195,196], genotoxicity assessment (i.e., BlueScreen, Ames, micronucleus assay) [160,197], and metabolite identification using human and preclinical species microsomes will aid in prioritization of compounds with limited reactive metabolite potential [198]. Further, covalent binding assessment with radiolabel can confirm protein binding and in combination with knowledge of absolute daily dose can predict potential DNA or protein reactivity based on established thresholds. Despite a robust read-across and weight of evidence approach, including early AhR-specific tiered screening assays, small molecule, and newer modality programs would likely require a 6-month transgenic (Tg.rasH2) mouse and 2-year rat bioassay study to assess carcinogenicity and support approval based on designation of the AhR as a susceptibility gene across various cancers and established procarcinogen action of specific AhR ligands (e.g., benzo(a)pyrene).

Prominent target organs for AhR-dependent toxicity liabilities reported within preclinical animal models and observed within human populations (e.g., skin, liver, and reproductive organs) warrant thorough and early evaluation within an AhR agonist program. Ad-hoc in vitro assays designed to address organ- and/or mechanism-specific safety concerns and subacute repeat-dose in vivo exploratory toxicity studies can provide critical information regarding potential off-target and modality-specific risks. For example, evidence of ligand- and AhR-dependent inhibition of ER-α or AR responses as well as developmental and reproductive toxicity concerns [199] warrant early assessment of biomarkers and gene transcriptomic signatures along with in vivo DART studies to evaluate AhR activation leading to cross species concordance of reproductive and developmental effects in men, women, and pediatric populations. In cases where endocrine disruption may be a concern, in vitro promoter–reporter assays with constructs containing ER-α or AR consensus binding sequences may be utilized as a screening tool. Additional immunotoxicity assays (e.g., complement assay) and cytokine/chemokine biomarker evaluation will also be needed to evaluate AhR-dependent immunomodulation. Lastly, considering the context-specific nature of ligand-dependent AhR activation and probable diverse gene signatures, thorough understanding of the patient population (e.g., age, race, gender, diet, lifestyle and co-morbidities) will aid in identifying predictive in vitro and in vivo models for de-risking AhR-dependent on- and off-target toxicities for a specific AhR-targeted molecule.

Despite high dose levels within preclinical species and long-term treatment durations, examination of approved drugs that activate the AhR across a wide variety of therapeutic areas revealed no overt toxicities synonymous with sustained dioxin-like AhR activation, and in fact, some of the compounds demonstrated anti-carcinogenic activity (Figure 4B,C) [200,201,202]. Data supporting that modulation of the AhR does not always present with an overt spectrum of DLC toxicities, and similar to other challenging drug targets (e.g., nuclear hormone receptors), this suggests that evaluation of the AhR using classic small molecule drug development paradigms can be an effective de-risking strategy. Noteably, acceptable risk levels across oncology and non-oncology programs will differ and AhR target modulation for inflammatory GI indications may require additional mechanistic investigation and early hazard identification screening efforts to support confidence in safety. Furthermore, challenges surrounding unknown risks pertaining to biotransformation of AhR agonists in the presence of environmental and dietary AhR ligands and whether chronic exposure of structurally diverse AhR ligands results in carcinogenicity requires further investigation. As outlined in previous sections, establishment of thresholds for AhR-dependent genes known to be involved in carcinogenic pathways (e.g., *CYP1A1/1A2*), physicochemical properties, and pharmacokinetic parameters (e.g., half-life, bioaccumulation, and AhR potency) will need to be front-loaded within an AhR agonist program to prioritize clean compounds. Utlimately, de-risking strategies for AhR programs will differ depending on the disease indication, modality, treatment duration, and patient population; however, addressing key AhR-dependent safety liabilities early has the potential to enable development of therapeutics targeting the AhR.

## 5. Summary and Conclusions

The stigma surrounding AhR target modulation originates from exhaustive research focused on mechanisms of dioxin-dependent AhR activation and downstream toxicity. The notion of the AhR as the “dioxin receptor” is further fueled by epidemiological studies that evaluated dioxin-exposed populations and reported increases in rates of morbidities and mortalities. The new age role of the AhR as an immunomodulatory agent has ignited novel research into beneficial roles of the AhR across various diseases, including inflammatory GI indications. The AhR has been shown to play a protective role within the gut by mediating immune homeostasis and maintaining barrier integrity, and several therapeutic modality approaches have been developed to target the AhR pathway to combat inflammatory bowel disease. Despite this advancement, AhR drug development is still limited by safety concerns; however, a safety-by-design approach to systematically identify and de-risk potential liabilities can empower AhR drug development programs. Novel chemical modalities targeting the AhR will also require a unique set of de-risking strategies to dial out toxicities. Thus, design, development, and implementation of robust in silico and in vitro tiered screening approaches as well as in vivo toxicity studies for AhR target modulation will not only enhance our understanding in the mechanism of action but will also build confidence in de-risking safety liabilities for inflammatory GI indications.

## Data Availability

Not applicable.
